# Short-sighted decision-making by those not vaccinated against COVID-19

**DOI:** 10.1038/s41598-022-15276-6

**Published:** 2022-07-13

**Authors:** Julia G. Halilova, Samuel Fynes-Clinton, Leonard Green, Joel Myerson, Jianhong Wu, Kai Ruggeri, Donna Rose Addis, R. Shayna Rosenbaum

**Affiliations:** 1 York University, 4700 Keele St., Toronto, ON M3J 1P3 Canada; 2 Baycrest Hospital, Toronto, Canada; 3 Washington University in St. Louis, St. Louis, USA; 4 Columbia University, New York, USA; 5 University of Toronto, Toronto, Canada; 6 The University of Auckland, Auckland, New Zealand

**Keywords:** Human behaviour, Predictive markers

## Abstract

Widespread vaccination is necessary to minimize or halt the effects of many infectious diseases, including COVID-19. Stagnating vaccine uptake can prolong pandemics, raising the question of how we might predict, prevent, and correct vaccine hesitancy and unwillingness. In a multinational sample (N = 4,452) recruited from 13 countries that varied in pandemic severity and vaccine uptake (July 2021), we examined whether short-sighted decision-making as exemplified by steep delay discounting—choosing smaller immediate rewards over larger delayed rewards—predicts COVID-19 vaccination status. Delay discounting was steeper in unvaccinated individuals and predicted vaccination status over and above demographics or mental health. The results suggest that delay discounting, a personal characteristic known to be modifiable through cognitive interventions, is a contributing cause of differences in vaccine compliance.

## Introduction

COVID-19 and its variants have had debilitating consequences to human life—both direct and indirect—that are likely to reverberate well beyond the current pandemic. To prevent severe illness and death, and alleviate hospital burden, extreme mitigation measures have been imposed, including lockdowns, quarantines, physical distancing, mask-wearing, and the rapid, wide-scale deployment of safe and effective vaccines for SARS-CoV-2. Widespread vaccination has been critical to containing the COVID-19 pandemic^1^ as well as other infectious diseases^2^, but it is threatened by vaccine hesitancy and resistance^3,4^. It is not enough to rely on predictive modeling of COVID-19 spread and vaccine uptake to guide behavioral change^3^; identifying actual behavioral markers of vaccine hesitancy and unwillingness is the crucial next step to reduce the severity and spread of COVID-19^5^, particularly given continued emergence of COVID-19 variants. Greater insight into the decision-making processes involved in vaccination choices can lead to strategies to better align behavior with medical and public health recommendations^6^.

Mounting evidence shows that, despite the unprecedented swiftness in development, approval, and deployment^7^, COVID-19 vaccines are largely safe and effective in protecting individuals from serious illness^8^, even in the face of novel variants of concern^9,10^, and that waning immunity can be addressed by an additional (booster) dose^11,12^. The UN’s global call to distribute primary doses widely and equitably attests to the international acceptance of COVID-19 vaccines^13^. Nevertheless, both the need for additional doses and the emergence of variants of concern that are less responsive to existing vaccines pose threats to vaccine acceptance. Future uncertainties surrounding vaccination strategy may further intensify ‘anti-vax’ attitudes^14^. The picture is complicated by demographic variables that can influence vaccine status: Older age, advanced education, and higher income increase both the likelihood that an individual has access to vaccines and that they will choose to be vaccinated^4,15,16^. Likewise, factors amplifying actual or perceived risk of COVID-19 exposure and infection, including living in geographic locales with higher case/death rates and working on the frontline in essential roles, may propel individuals to seek vaccination^17,18^.

The clear and present need for broad and sustained vaccine uptake to end the COVID-19 pandemic and prevent future outbreaks has fueled scientists’ and public health officials’ drive to identify and promote ways to increase vaccination rates. Methods to induce behavioral change, including public education campaigns, reminders, attention to societal impact, and monetary incentives, all have shown some success^4,7,19^, at least in the short term. To address ongoing and future challenges associated with COVID-19 and other infectious disease threats, there is an urgent need for solutions that produce longer-term behavioral change with respect to vaccination unwillingness and hesitancy. The first step is to identify target behavioral variables that predict individuals’ vaccination decisions.

A promising behavioral economic measure of decision-making is *delay discounting*, which assesses the tendency to forgo larger, delayed rewards in favor of smaller, immediate rewards. Each individual has an indifference point where the value of a future reward is sufficiently large as to offset the delay until gratification^20^. The higher the indifference point, the more an individual is taking future benefits into account and the greater the subjective value of the delayed reward; the lower the indifference point, the lower the subjective value and the greater the short-sighted bias in decision-making. Steep discounting of delayed rewards (evidenced by lower indifference points) is associated with many negative outcomes, including financial instability^21^ and problematic health behaviors^22,23^, both of which have intensified since the start of the pandemic^24–26^. Critically, for public health purposes, delay discounting is modifiable. For example, cueing individuals to imagine specific future events has proven effective in reducing the degree of discounting in diverse populations^23,27–29^.

**Figure 1:**
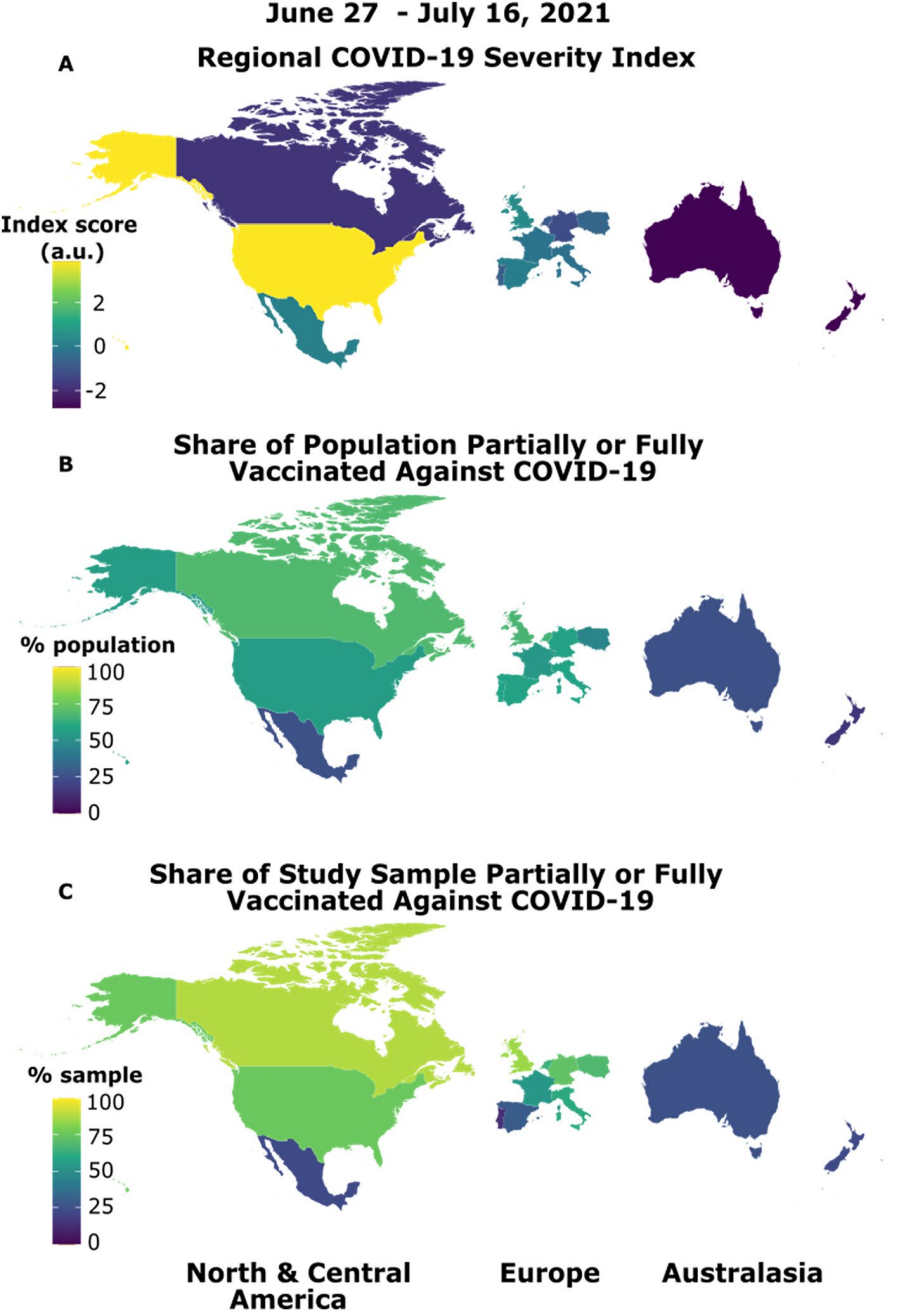
Pandemic and vaccine situations varied across our multinational sample at the time of testing. The R package "maps" was used to visualize regional differences on the COVID-19 Regional Severity Index and population and study sample vaccine situations (https://cran.r-project.org/web/packages/maps/index.html). (**A**) The Regional COVID-19 Severity Index is a nation’s component score (in arbitrary units, a.u.) from a principal component analysis of weekly COVID-19 cases/death rates, total cases/deaths since the first week of 2020, and population-adjusted total cases/deaths per 100,000. These nation-specific data were extracted from the European Centre for Disease Prevention and Control COVID-19 statistics^30^ for each participant based on the week they completed the study. (**B**) The share of each nation’s population who were partially or fully vaccinated (i.e., one or more doses) against COVID-19, shown as the average percentage across our testing window; data were extracted from^31^. These data show lower proportions (15%) in countries only beginning vaccine roll-out (e.g., New Zealand) to almost 70% of the population in countries with earlier access to vaccines (e.g., United Kingdom, United States) and/or rapid uptake (e.g., Canada). (**C**) The share of participants from each country who were partially or fully vaccinated against COVID-19 (range 13% to 88%). Our sample was generally representative of population rates; the difference between sample rates (**C**) and population rates (**B**) for each country are plotted in Fig. S1.

Here, we combine a large, multi-nation sample with a highly sensitive online delay discounting task that uses an adjusting-amount procedure to determine an individual’s short-sighted bias in decision-making. We strategically sampled from a range of industrialized nations across Australasia, Europe, and North America that varied in local severity of the pandemic due to variants of concern at the time of testing (June 27 to July 16, 2021)^5^. This was confirmed across the 13 nations we sampled by examining real-time pandemic severity statistics linked to participation dates (see Fig. 1A). Data collection took place after primary vaccines were deployed and before booster doses were introduced. Average rates of vaccination (partial and full, combined) ranged from 15 to 69% across nations during our testing window (Fig. 1B). Our key analyses demonstrate that, even after accounting for country-level differences as well as demographics and mental health variables, short-sighted decision-making emerges as a unique predictor of being unvaccinated against COVID-19, demonstrating its promise as a predictor of vaccine unwillingness and as a target for interventions.

## Results

Recruitment was conducted through an online platform (Prolific.co) from June 27, 2021 to July 16, 2021. Data from 4,452 participants were analyzed: 1,566 were fully vaccinated (two doses or one dose for J&J/Jansen), 1,033 were partially vaccinated (e.g., one dose for Moderna/Pfizer); 1,440 were unvaccinated but planning to be, and 413 were unvaccinated and not planning to be. The groups were combined into a binary vaccination status variable (i.e., vaccinated vs. unvaccinated) to capture participants’ vaccination decisions. Figure 1C shows the proportion of our sample from each country who were vaccinated.

Participants indicated their gender, age, highest level of education, and whether they worked in an occupation deemed essential during the pandemic. Given the multinational sample, income was assessed as participants’ rating of their income as low, average, and high incomes in their own region/country on a 100-point scale^32^. A psychological distress index was included to control for anxiety and depressive symptoms that may interact with other variables, including delay discounting^22^, in the analysis. Delay discounting was measured using an established intertemporal choice procedure^27,29^. On each of 42 trials, participants decided between a larger, later hypothetical reward (e.g., $2,000 one month from now) and a smaller, immediate reward (e.g., $1,000 today). A staircase procedure adaptively determined the choice amounts presented on each trial based on prior responding. Given the existence of multiple discounting models^20^, a well-established, theoretically neutral measure, Area-Under-the-Curve (AuC), was used to assess biased decision-making^33^. Descriptive statistics for all key predictors in our analyses (as well as gender) are presented in Table 1 by vaccination status.

**Table 1:** Participant characteristics by vaccination status.

	Unvaccinated (n = 1853)	Vaccinated (n = 2599)
Gender (% female/male/non-binary)	45/53/1	53/46/1
Mean age in years (SD)	27.96 (8.79)	32.22 (11.48)
Highest level of education (% secondary/undergraduate/postgraduate)	32/52/16	28/50/22
Mean rating of relative income^a^ (SD)	36.31 (23.8)	40.39 (23.97)
Essential worker (% yes)	15	27
Mean psychological distress index score (SD)	0.11 (1.89)	− 0.08 (1.89)
Delay discounting (AuC)	0.38 (0.25)	0.41 (0.25)

^a^100-point scale, where 0 = low, 50 = medium, and 100 = high relative to others in the participants’ country/region.

*AuC* area-under-the-curve (range, 0–1), *undergrad* undergraduate degree or professional equivalent, *postgrad* postgraduate degree (e.g., Masters, PhD), *SD* standard deviation.

Our key analysis determined the unique contribution of discounting delayed rewards to predicting the odds of being vaccinated after accounting for other variables. A multilevel logistic regression model was constructed with vaccination status (unvaccinated vs. vaccinated) as the outcome variable, and age, education level, income, distress index, essential worker status, and AuC as predictors. To account for possible systematic differences across countries (e.g., COVID-related severity, population vaccination rates, government response), each participant’s vaccination status (Level 1) was nested within country (Level 2; intraclass correlation, *ICC* = 0.30). The model accounted for significantly more variance in the data compared to an unconditional intercept-only model, *χ*^2^*(6)* = 221.54, *p* < 0.001. Results show that the tendency to choose larger future rewards over smaller immediate ones significantly increases the odds of being vaccinated above and beyond the influence of other variables in the model (*p* < 0.001; Table 2). All of these variables were significantly associated with the likelihood of being vaccinated (*p* values < 0.008), with the exception of the psychological distress index (*p* = 0.98), which was not significant as a unique predictor of vaccination status.

**Table 2:** Results of the multilevel logistic regression model predicting vaccination.

Fixed effects	Estimate	SE	*z*	*p*	OR	95% CI
Intercept	− 2.50	0.42	− 5.98	< 0.001	0.08	[0.04, 0.19]
Age	0.04	0.01	8.56	< 0.001	1.04	[1.02, 1.04]
Education level	0.27	0.06	4.89	< 0.001	1.31	[1.18, 1.46]
Income	0.004	0.002	2.66	0.008	1.00	[1.00, 1.01]
Essential worker	0.58	0.10	5.94	< 0.001	1.79	[1.48, 2.17]
Psychological distress	0.001	0.02	0.02	0.98	1.00	[0.96, 1.04]
Delay discounting (AuC)	0.53	0.15	3.56	< 0.001	1.70	[1.27, 2.28]

Random Effects	Estimate	SD				
Intercept error variance (country)	1.54	1.24				

*AuC* area-under-the-curve, *CI* confidence interval, *OR* odds ratio, *SD* standard deviation, *SE* standard error.

## Discussion

**Figure 2:**
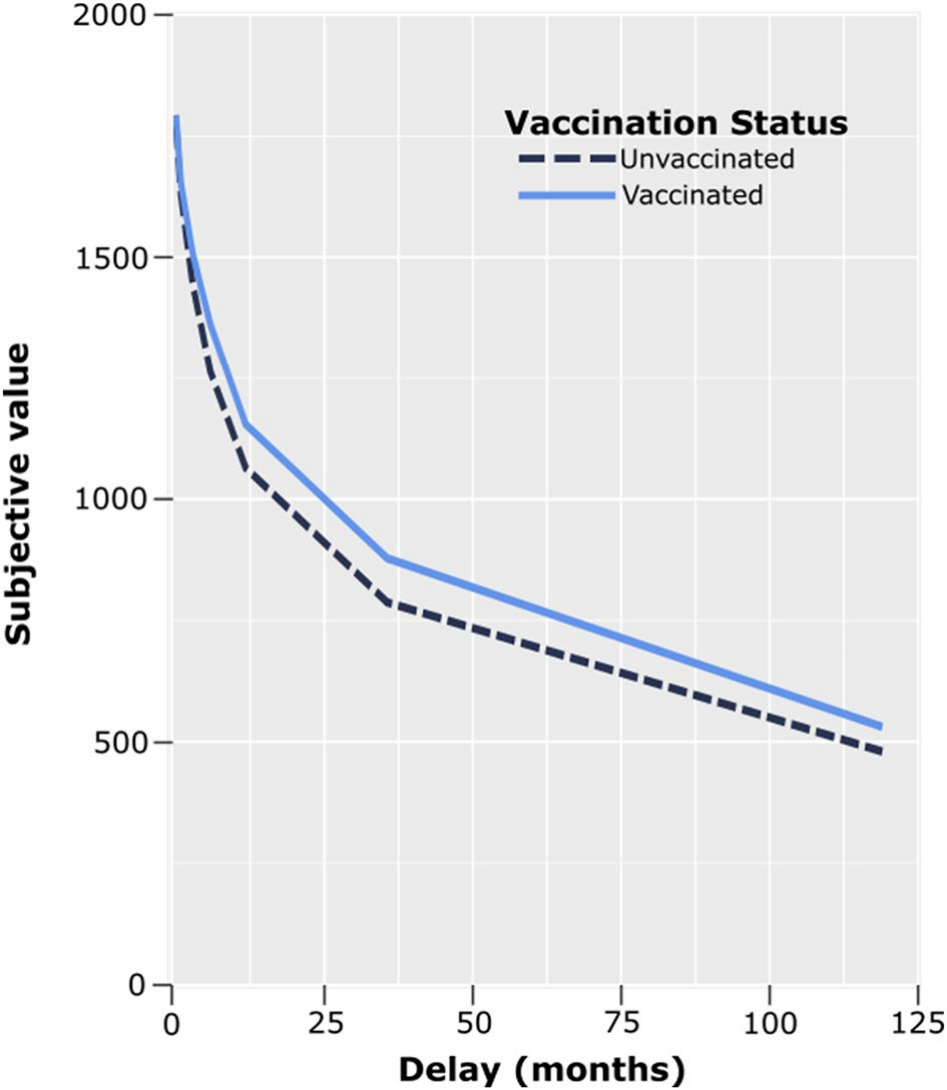
Discounting curves in vaccinated and unvaccinated participants. Subjective value (mean indifference point) of the $2,000 delayed reward as a function of the delay to its receipt. Area-under-the-Curve (AuC) was used as a measure of delay discounting. Unvaccinated individuals on average tended to discount future rewards more steeply (i.e., have smaller AuCs) than vaccinated individuals.

We show that COVID-19 vaccination is predicted by a greater propensity to choose larger, future rewards over smaller, immediate rewards, as indicated by shallower delay discounting (Fig. 2). This finding emerges across multiple countries that varied in pandemic severity and vaccination rates at the time of testing. Discounting explains unique variance over and above other predictors of vaccine acceptance, including higher age, education level, and income level, as well as employment as an essential worker. Lack of protection against COVID-19 places individuals and societies at sustained risk of illness and death, prolonging a safe return to pre-pandemic life. Identifying delay discounting as a source of vaccine non-compliance provides an avenue for inducing positive behavioral change in the face of global threats to health and safety.

The current results are in line with previous findings showing that steeper delay discounting is a key predictor of numerous health-related issues, including obesity, addictive behaviors, and anxiety and mood disorders^23^, many of which are exacerbated by pandemic conditions^25,26^. The extent to which delay discounting is a predictor of compliance with pandemic-mitigating behaviors other than vaccination (e.g., physical distancing, mask-wearing) is less clear^34–36^. Seemingly weak or contradictory findings may reflect the influence of confounding factors, such as psychological distress, which tends to be positively related to delay discounting^37^. Given the association between psychological distress and delay discounting^23^, it is perhaps not surprising that psychological distress did not predict vaccination status after controlling for other variables.

Although our multi-national sample spanned three global regions, it was nevertheless limited to industrialized countries that, with the exception of Mexico, fall under the Western Educated Industrialized Rich and Democratic (WEIRD) designation^38^. As vaccine availability becomes more widespread globally, this work should be expanded to determine the utility of delay discounting as a marker of vaccine acceptance in non-industrialized countries, particularly given the considerably different government responses, vaccine access, and/or economic situations. We recognize that in addition to the factors examined here, vaccination decisions also are influenced by individual differences in political ideology, respect for authority, vaccine literacy, trust in vaccine information provided by government public health agencies, and trust in science more generally^39^. In contrast to these factors, which can be challenging to measure, discounting is a simple, well-established measure of decision-making that the present findings show is a conspicuous indicator of vaccination choices.

Discounting the value of future monetary rewards parallels opting for the smaller, immediate benefits of not getting vaccinated (e.g., avoiding initial side effects) versus the longer-term benefits of vaccination (e.g., immunity to COVID-19, increased social interactions). That is, steep discounting is a proxy for short-sighted decision-making. Sustainable policy applications to encourage vaccine uptake should directly address short-sighted decision-making in the form of steep discounting through use of established methods that make future consequences more salient^6,23,27,29^, supplemented by use of immediate, modest monetary incentives, which have shown some success^3^. Such interventions are critical as even greater disparities in vaccine acceptance are expected if and when new variants of concern emerge, boosters or modified vaccines are recommended and available, and vaccinations against other infectious diseases regain attention. Turning to the behavioral sciences to understand the decision-making process underlying vaccine acceptance is crucial when the outcome of the decision has the potential to harm oneself and other people.

## Materials and methods

### Participants

Using Prolific’s built-in inclusion/exclusion function, the study was available only to users meeting the following inclusion criteria: aged 18 years or older, fluent in English, currently residing in one of 14 target countries across North America, Europe, Australasia, and Africa, and free from neurological impairments or learning disabilities. All 5,193 participants provided informed consent and received monetary compensation at a rate recommended by Prolific. Data from 320 individuals were excluded from the analyses: 17 due to failure to meet inclusion criteria (e.g., residing in a non-targeted country); 176 due to non-completion of the survey; 86 due to not reporting vaccination status; and 41 due to responding incorrectly to more than one attention check item (see below). Data from 421 participants from South Africa also were excluded due to challenges in obtaining reliable COVID-related metrics at the population level (e.g., COVID-19 case rates, vaccination rate), substantial differences in government response compared to other countries included in the analyses^40^, and very low vaccination rate (only 23 participants from South Africa in our sample reported being vaccinated). The study was approved by the York University and Baycrest Research Ethics Boards for research with human participants (REB #08–57), and all research was conducted in accordance with the Declaration of Helsinki.

### Materials

All data were collected in an online Qualtrics survey environment. Participants completed a survey that included the following sections (along with other measures not reported here):

#### Delay discounting task

In this intertemporal choice procedure^27,29^, participants viewed pairs of monetary amounts and were asked to choose between a smaller, immediate reward, which varied between trials, and a larger, delayed reward of $2,000. Participants were asked to make six choices at each of seven delays (waiting 1 week, 1 month, 3 months, 6 months, 1 year, 3 years, and 10 years before receiving the $2000 reward). An iterative, adjusting-amount procedure was used in which the amount of the immediate reward was increased or decreased based on the participant’s previous choice at that delay, converging on the amount of the immediate reward equivalent in subjective value to the delayed reward. The first adjustment was half of the difference between the immediate and delayed amounts presented on the first trial, with each subsequent adjustment being half of the preceding adjustment. For example, in the condition where a future reward of $2000 could be received in 3 years, the first choice presented to the participants would be “$1000 right now or $2000 in 3 years.” If the participant chose “$2000 in 3 years,” the choice on the second trial would be “$1500 right now” or “$2000 in 3 years.” If the participant then chose “$1500 right now”, the choice on the third trial would be “$1250 right now or $2000 in 3 years.” Following the sixth and final trial of each condition, the subjective value of the delayed reward was estimated as the amount of the immediate reward that would be presented on a seventh trial. Degree of discounting was measured by examining the relation of subjective value to delay of reward and computing AuC, a single, theoretically neutral measure of discounting^33^.

#### Demographic questionnaire

Participants completed a demographic questionnaire that included items probing: country of residence, age, gender (female/male/non-binary), highest level of education obtained (secondary schooling/undergraduate degree or professional equivalent/postgraduate degree), and essential occupation (yes/no). Occupations deemed essential during the pandemic are those supplying critical services: government; health and safety (e.g., healthcare, emergency response); utilities (e.g., water, energy, sanitation, transport, communications); food (e.g., supermarkets); and manufacturing. A measure of relative income was used: participants estimated their current income on a sliding scale (0–100) marked by points representing low (0), average (50), and high (100) incomes in their own country/region^SPS:refid::bib3232^.

#### Mental health questionnaires

Presence and severity of anxiety and depressive symptoms were assessed with the Generalized Anxiety Disorder 7-item (GAD-7) scale^41^ and the Patient Health Questionnaire 9-item (PHQ-9) scale^42^. Participants rated the frequency of symptoms experienced over the past two weeks on a four-point scale (0 = not at all; 3 = nearly every day). For each scale, a total score was computed, where higher scores reflect more severe symptoms. Total scores from these measures were standardized and then summed to create a psychological distress index.

#### Attention checks

Three items from the Conscientious Responder Scale^43^ were included at select points within the survey to identify random responders (e.g., “To answer this question, please choose option three, neither agree nor disagree.").

### Analyses

#### Regional COVID-19 severity index

Weekly cases/deaths, cumulative total cases/deaths, and cumulative cases/deaths per 100,000 people were extracted from the ECDC COVID-19 statistics^25^ for each participant on the week of survey completion for the country in which they resided. The dimensionality of these data was reduced using principal components analysis (PCA) with the aim of isolating a single component reflecting shared variance across the different COVID-19 severity statistics. PCA is a multivariate technique used to reduce data dimensionality whilst maximally maintaining its variability. In the present study, PCA was conducted in R using the *prcomp()* function of the *stats* package and results were extracted using the package *factoextra*^44^. The data were first mean-centered and scaled (i.e., mean = 0, standard deviation = 1), rendering the analysis equivalent to running PCA on the correlation matrix. The *prcomp()* function PCA is performed using the singular value decomposition method and results in orthogonal principal components (PC) that maximally retain the correlations among individuals. PCs with an eigenvalue (λ) > 1 were considered reliable. The Regional COVID-19 Severity Index comprised the component scores from PC1, which accounted for 63.5% of variance in the data and corresponded to shared variance across all ECDC variables (λ = 3.81). Higher individual component scores reflected greater regional severity of COVID-19.

#### Multilevel logistic regression

This model was constructed using R packages *lme4*^45^ and *lmerTest*^46^, with the outcome variable (participants’ vaccination status; Level 1) nested within country (Level 2), and with age, education level, income, essential worker status, psychological distress index, and AuC as predictors. The model was estimated using maximum likelihood with Laplace approximation.

#### Data visualization

Plots were constructed using R package *ggplot2*^47^. Geomapping of the Regional COVID-19 Severity Index and vaccination rates was achieved using the *R* package *maps*^48^ (license agreement: https://cran.r-project.org/web/licenses/GPL-2).

## Supplementary Information

Supplementary Information.

## Data Availability

Anonymized, raw, and cleaned data as well as code necessary to reproduce the results and figures, have been deposited in a public repository hosted by the Open Science Framework (https://osf.io/ms8w2/?view_only=1a93126ca71447e4bde4bd17f4e9a90b).
