# Supplementary material for: Short-sighted decision-making by those not vaccinated against COVID-19
Source: Sci Rep. 2022 Jul 13;12:11906. doi: 10.1038/s41598-022-15276-6 (PMC9277980; doi:10.1038/s41598-022-15276-6)
Supplement: Supplementary file 1 — Supplementary Information. [file 41598_2022_15276_MOESM1_ESM.docx]

**SUPPLEMENTAL MATERIALS**

**SUPPLEMENTAL RESULTS**


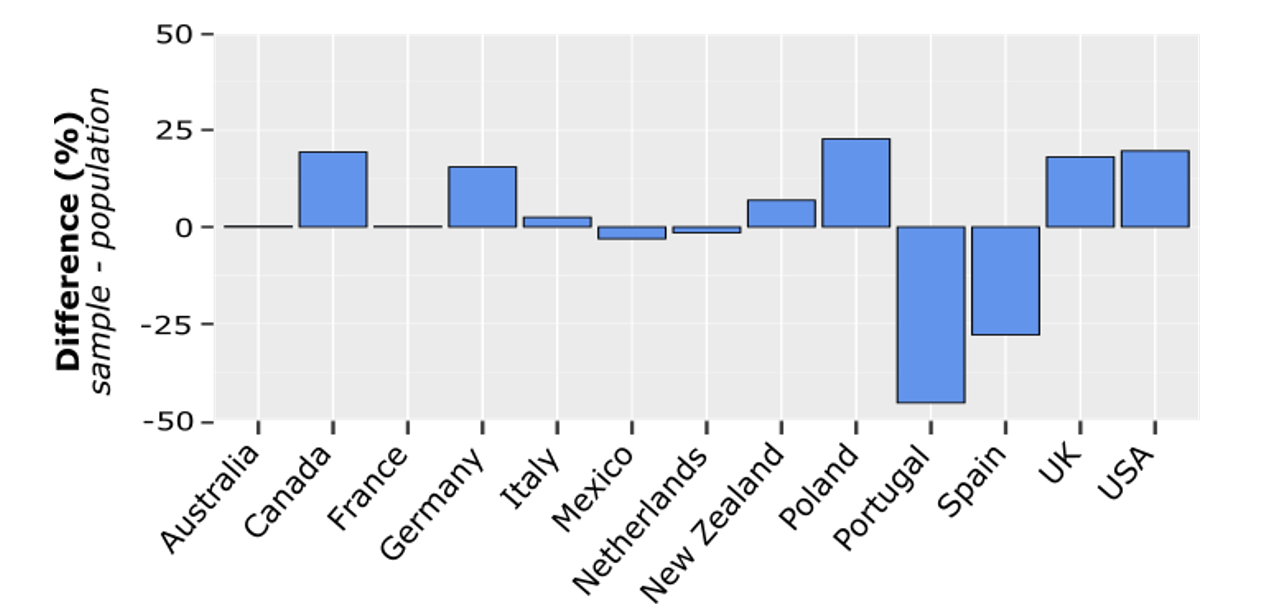


**Figure S1. Differences in population and sample level vaccination rates.** The percentage of participants sampled from each country who were vaccinated (either partially or fully) against COVID-19 was contrasted with the population rate of vaccination for that country at the time of data collection. Positive percentage difference values indicate a higher vaccination rate in our sample; negative percentage difference values indicate a higher vaccination rate in the population. For the majority of countries, the vaccination rate captured in our sample is representative of the reported population vaccination rate, with differences of less than 25 percentage points. The largest differences in vaccination rates between our sample and that reported for the population were in Portugal and Spain, on the order of 25-45 percentage points. To ensure this difference did not influence our results, the logistic multilevel regression was rerun excluding data from these two countries in the data set, and the main findings remained unchanged. UK=United Kingdom; US=United States.

**Table S1. Descriptive Statistics by Country**

| **Country (n)** | **Age** | | **Income** | | **Distress** | | **AuC** | |
| --- | --- | --- | --- | --- | --- | --- | --- | --- |
|  | **UV** | **V** | **UV** | **V** | **UV** | **V** | **UV** | **V** |
| Australia (333) | 31.31 (10.16) | 38.56  (13.49) | 35.77 (25.23) | 47.49 (21.85) | -0.20 (1.78) | -0.63 (1.76) | 0.38 (0.25) | 0.43 (0.25) |
| Canada (538) | 31.35 (10.13) | 32.15 (11.30) | 32.78 (22.79) | 39.74 (23.64) | 0.32 (2.00) | -0.01 (1.93) | 0.38 (0.25) | 0.42 (0.25) |
| France (96) | 27.47 (7.61) | 30.20 (10.77) | 34.18 (26.86) | 31.31 (27.86) | 0.11 (1.91) | 0.21 (2.04) | 0.35 (0.20) | 0.45 (0.26) |
| Germany (125) | 31.91 (12.03) | 30.23 (8.71) | 31.06 (23.99) | 37.44 (24.13) | -0.41 (1.74) | -0.06 (1.70) | 0.35 (0.27) | 0.46 (0.23) |
| Italy (396) | 27.45 (7.54) | 29.02 (8.31) | 29.34 (21.16) | 36.22  (21.48) | 0.46 (1.96) | 0.24 (1.84) | 0.39 (0.26) | 0.43 (0.26) |
| Mexico (291) | 25.79 (5.23) | 32.52 (8.99) | 42.82 (21.69) | 52.13 (20.48) | 0.31 (1.92) | -0.44 (1.80) | 0.28 (0.24) | 0.35 (0.25) |
| New Zealand (134) | 24.40 (6.92) | 29.00 (10.39) | 36.21 (26.69) | 41.02  (24.75) | 0.04 (1.76) | -0.29 (1.60) | 0.46 (0.26) | 0.49 (0.24) |
| The Netherlands (104) | 33.4 (11.95) | 33.65 (11.65) | 40.46 (23.24) | 56.43 (22.51) | -0.54 (1.45) | -1.06 (1.65) | 0.40 (0.22) | 0.46 (0.25) |
| Poland (411) | 23.87 (6.24) | 23.67 (6.33) | 37.87 (22.43) | 38.50 (24.50) | 0.30 (1.80) | 0.39 (1.86) | 0.38 (0.25) | 0.37 (0.25) |
| Portugal (403) | 24.31 (5.20) | 34.00 (10.76) | 34.24 (24.31) | 46.22 (24.41) | 0.30 (1.93) | -0.06 (1.85) | 0.42 (0.27) | 0.42 (0.29) |
| Spain (288) | 27.23 (7.57) | 42.00 (10.42) | 34.77 (23.20) | 49.58 (20.33) | -0.05 (1.76) | -0.66 (1.74) | 0.39 (0.23) | 0.42 (0.26) |
| UK (602) | 29.05 (9.91) | 34.77 (11.86) | 36.16 (23.80) | 40.57 (23.66) | -0.08 (1.86) | -0.11 (1.89) | 0.39 (0.22) | 0.40 (0.23) |
| USA (731) | 33.51 (10.45) | 33.90 (12.11) | 40.54 (23.75) | 39.59 (24.73) | 0.02 (2.14) | -0.21 (1.93) | 0.37 (0.26) | 0.41 (0.26) |

Note. AuC=Area-under-the-Curve; UK=United Kingdom; US=United States; UV=Unvaccinated; V=Vaccinated.
